# Exploring the Clinical Features, Management of Hypertension, and Predictors of Severity in Hospitalized Hypertensive COVID-19 Patients

**DOI:** 10.7759/cureus.61356

**Published:** 2024-05-30

**Authors:** Narendar Kumar, Syed Azhar Syed Sulaiman, Furqan K Hashmi, Ahmed Noor, Rabbiya Ahmad, Ali Qureshi, Faheem Jhatial, Siti Maisharah Sheikh Ghadzi

**Affiliations:** 1 Clinical Pharmacy, School of Pharmaceutical Sciences, Universiti Sains Malaysia, Pulau Pinang, MYS; 2 Pharmacy Practice, Faculty of Pharmacy, University of Sindh, Jamshoro, PAK; 3 Pharmacy, University of the Punjab, Lahore, PAK; 4 Cardiology, Indus Hospital & Health Network, Karachi, PAK; 5 Pharmacy Services, Indus Hospital & Health Network, Karachi, PAK

**Keywords:** clinical feature, severity, hypertension, covid-19, sars-cov-2

## Abstract

Background

Hypertension significantly contributes to the severity, prolonged hospitalization, the need for intensive care, and mortality of COVID-19 patients. However, the data is still evolving. This study investigated the predictors of severity among hypertensive COVID-19 patients.

Methodology

This cohort study included 333 hospitalized hypertensive COVID-19 patients at the Indus Hospital, Karachi, Pakistan, from April 2021 to October 2021. The study evaluated the clinical features, antihypertensive therapy, and predictors of severity. A multivariable binary logistic regression model was used to determine severity predictors using IBM SPSS Statistics for Windows, Version 27.0 (Released 2020; IBM Corp., Armonk, NY, USA).

Results

The majority of hypertensive COVID-19 patients were females (54.7%), aged <65 years (55.8%), and coexisted with diabetes mellitus (56.5%). The independent predictors of severity were male (aOR 2.65, 95% CI, 1.08-6.51; p < 0.033), fever (aOR 3.52, 95% CI, 1.24-9.92; p = 0.017), shortness of breath (aOR 4.49, 95% CI, 1.73-11.63; p = 0.002), oxygen saturation (<90%) (aOR 87.39, 95% CI, 19.15-398.75; p < 0.001), and D-dimer (>0.5 mcg/ml) (aOR 3.03, 95% CI, 1.19-7.71; p = 0.020).

Conclusions

Our study concluded that males with fever before admission, shortness of breath, lower oxygen saturation, and elevated D-dimer are the predictors of severity among hypertensive COVID-19 patients.

## Introduction

COVID-19 was discovered when some patients with pneumonia of unidentified origin were reported in Wuhan, China in late December 2019 [1]. In Pakistan, the first laboratory-confirmed case of SARS-CoV-2, subsequently referred to as COVID-19, was reported on February 26, 2020. Following this, an appreciable number of positive cases were reported throughout the country in successive waves. As of August 2, 2023, Pakistan has recorded over 1.58 million positive cases and 30,000 deaths [2].

Pakistan has grappled with five waves of COVID-19, and each wave is driven by different SARS-CoV-2 variants, with a notable difference in the severity of COVID-19 observed during these waves [3]. Despite initiating vaccination campaigns in February 2021, many people were hesitant to get vaccinated against SARS-CoV-2 [4], which was influenced by their knowledge and beliefs toward COVID-19 since its outbreak in the region [5]. Consequently, vaccination rates remained low in the country, and in 2021, the country underwent the third and fourth waves of SARS-CoV-2, which were characterized by more transmissible SARS-CoV-2 variants such as Delta and Omicron. The Delta variant led to high hospitalization rates, severe illness, increased admission to intensive care units, and fatalities in the country [6].

According to the data from the World Health Organization, approximately 29% of Pakistani people died due to cardiovascular diseases [7]. Research indicates that patients with comorbid illnesses may experience severe clinical outcomes when contracting COVID-19. However, patients with similar characteristics and clinical conditions may experience diverse clinical outcomes among COVID-19 patients, including severe illness and higher mortality rates [8]. Additionally, hypertension (HTN) has been a high-risk factor for poor outcomes [9], including severity and mortality among COVID-19 patients, as well as intensive care unit admission and prolonged hospital stay [10]. Since HTN is a frequently observed problem in adults, especially in elderly populations, it is a prevalent comorbidity in COVID-19 patients [11]. However, the extent and predictors of severity among hypertensive COVID-19 patients during recent waves of COVID-19 with different SARS-CoV-2 variants have not been studied well. Therefore, exploring the predictors of severe COVID-19 among hypertensive patients is of paramount importance to reduce the mortality rates. The successful implementation of such a predictive model could have significant implications for public health issues, as it would increase our understanding of the progression of COVID-19 and help us determine disease severity, which may lead to fatal outcomes. This study aimed to explore the clinical characteristics and antihypertensive treatment and determine the predictors of severity among hypertensive COVID-19 patients.

## Materials and methods

Study design and setting

This single-center observational study was conducted at Indus Hospital, Karachi, Pakistan, during the third and fourth COVID-19 waves. All COVID-19 patients with preexisting HTN admitted to COVID-19 wards between April 1 and October 31, 2021 were enrolled using a non-probability purposive sampling technique.

Study population

Patients with preexisting HTN confirmed by their past medical history and infected with COVID-19 were included in this study.

Inclusion and exclusion criteria

Only patients aged 18 years and older with HTN and COVID-19 were included in the study. On the other hand, pregnant women, children under 18 years, and non-hypertensive COVID-19 patients were excluded from the study.

Research instruments and data collection

The data was accessed from the electronic medical records of the patients and recorded on a research instrument. The research instrument included information on patients’ demographic characteristics, such as age, gender, comorbidities, and smoking status. Moreover, clinical features, including baseline signs and symptoms, vital signs, laboratory reports (blood counts, liver function tests, cardiac and inflammatory markers), antihypertensive treatments, and final stage severity [12], were recorded on the research instrument. Patients with one or more symptoms, having more than 50% lung infiltrates, a respiratory rate greater than 30 breaths per minute, SpO2 ≤90% on room air, and an arterial oxygen partial pressure/fractional inspired oxygen (PaO2/FiO2) ratio <300 were considered severe. Patients with or without symptoms, having less than 50% lung infiltrates, a respiratory rate less than 30 breaths per minute, SpO2 ≤94% and >90% on room air, and an arterial oxygen partial pressure/fractional inspired oxygen (PaO2/FiO2) ratio >300 were considered non-severe.

Statistical analysis

The data was keyed into IBM SPSS Statistics for Windows, Version 27.0 (Released 2020; IBM Corp., Armonk, NY, USA). For normality testing, we used the Kolmogorov-Smirnov test of normality testing. Non-normally distributed continuous variables were expressed in medians and interquartile ranges, whereas categorical variables were expressed in numbers and percentages. A binary logistic regression model determined the predictors of severity using univariable and multivariable regression analysis. Statistical significance was considered at a p-value ≤0.05.

Ethical approval

This study was approved by the Institutional Review Board (IRB) of Indus Hospital & Health Network, Korangi Campus, Karachi, Pakistan (approval number IHHN_IRB_2021_06_008).

## Results

Demographic characteristics of patients

A total of 333 hypertensive COVID-19 patients were included in this study. Most of the patients were females (182; 54.7%), with a median (IQR) age of 62 (55-70) years and less than 65 years (186; 55.8%). Only 19 patients (5.7%) were smokers, and 27 (8.1%) were fully vaccinated against COVID-19. Moreover, diabetes mellitus was the most frequent coexisting comorbidity (187; 56.5%), followed by cardiovascular diseases (93; 27.9%), and chronic kidney disease (36; 10.8%), as described in Table 1.

**Table 1:** Demographic characteristics of patients (N = 333) ** Parkinson’s disease, epilepsy, arthritis, osteoarthritis, chronic liver disease, immune thrombocytopenia, systemic lupus erythematosus, and gout

Variables	N (%)	
		Gender		
Female	182 (54.7)	
Male	151 (45.3)	
Age (years), median (IQR)	62 (55-70)	
Age groups		
<65 years	186 (55.8)	
≥65 years	147 (44.2)	
Smoking status		
Nonsmoker	305 (91.6)	
Ex-smoker	9 (2.7)	
Smoker	19 (5.7)	
Vaccination status		
Not vaccinated	291 (87.4)	
Partially vaccinated	15 (4.5)	
Fully vaccinated	27 (8.1)	
Comorbidity		
Diabetes mellitus	188 (56.5)	
Cardiovascular disease	93 (27.9)	
Chronic kidney disease	36 (10.8)	
Asthma	13 (3.9)	
Benign prostate hyperplasia	10 (3.0)	
Hepatitis	10 (3.0)	
Hypothyroidism	9 (2.7)	
Chronic obstructive pulmonary disease	6 (1.8)	
Other**	20 (6.0)	

Baseline clinical characteristics of patients

The clinical signs and symptoms of COVID-19 patients are presented in Table 2. Most of the patients experienced fever (83.8%), shortness of breath (82.0%), productive cough (38.7%), generalized weakness (28.5%), and dry cough (16.8%) during the illness. However, a limited number of patients observed sore throat (4.2%), tiredness or fatigue (3.3%), and headache (2.1%). Regarding baseline vital signs, the patients’ median systolic blood pressure was 140 (123-156), while the diastolic blood pressure had a median value of 80 (70-89). The median heart rate was 95 (81-110), and the median respiratory rate was 30 (24-36). The median oxygen saturation level was 85 (76-90).

**Table 2:** Clinical characteristics of patients (N = 333) ** Constipation, chest congestion, flu, insomnia, nausea, palpitations, and loss of smell and/or taste

Variables	N (%)	
		Signs and symptoms		
Fever	279 (83.8)	
Shortness of breath	273 (82.0)	
Productive cough	129 (38.7)	
Weakness	95 (28.5)	
Dry cough	56 (16.8)	
Vomiting	40 (12.0)	
Malaise	36 (10.8)	
Chest pain	32 (9.6)	
Diarrhea	29 (8.7)	
Chills and rigors	23 (6.9)	
Abdominal pain	17 (5.1)	
Anorexia	29 (8.7)	
Altered level of consciousness	16 (4.8)	
Sore throat	14 (4.2)	
Tiredness	11 (3.3)	
Headache	7 (2.1)	
Drowsiness	8 (2.4)	
Other**	22 (6.6)	
Baseline vitals		
Systolic blood pressure, mmHg	140 (123-156)	
Diastolic blood pressure, mmHg	80 (70-89)	
Heart rate, beats/min	95 (81-110)	
Temperature, °F	98.4 (98.4-98.8)	
Respiratory rate, breaths/min	30 (24-36)	
Oxygen saturation, %	85 (76-90)	

Baseline laboratory characteristics of patients

The baseline laboratory characteristics of the patients are summarized in Table 3. The result shows that the median total leucocyte count was 11.3 (7.5-15.3) × 10^9^/L, slightly above the normal range’s upper limit, and the median percentage of neutrophils was very high, 84.3% (77.6-90.6%), which indicates the immune response to the viral infection or a severe inflammatory response to the virus. In contrast, the median percentage of lymphocytes was 9.2% (4.8-14.5%), and eosinophils 0.1% (0.0-0.3%) were lower than normal. The median value of serum bicarbonate was low, 20.6 (16.9-23.7) mEq/L.

**Table 3:** Baseline laboratory indicators (N = 333) ALP, alkaline phosphatase; ALT, alanine transaminase; GGT, gamma-glutamyl transferase; HS-cTnI, high-sensitivity cardiac troponin-I; INR, international normalized ratio; LDH, lactate dehydrogenase; PT, prothrombin time; RBG, random blood glucose; TLC, total leucocyte count

Variables	Normal range	Median (IQR)
Blood cells count		
Hemoglobin, g/dL	Male: 13.7-16.3; female: 11.1-14.5	12.3 (10.4-13.5)
TLC, × 10^9^/L	4-10	11.3 (7.5-15.3)
Neutrophils, %	40-70	84.3 (77.6-90.6)
Lymphocyte, %	20-45	9.2 (4.8-14.5)
Monocytes, %	02-10	4.9 (3.1-7.1)
Eosinophils, %	01-06	0.1 (0.0-0.3)
Basophils, %	0-1	0.4 (0.2-0.7)
Platelets, × 10^9^/L	150-400	235 (182-323)
Serum creatinine and electrolyte		
Creatinine, mg/dL	0.57-1.25	1.1 (0.81-1.83)
Calcium, mg/dL	8.4-10.2	8.2 (7.8-8.7)
Sodium, mEq/L	136-145	136 (132-140)
Potassium, mEq/L	3.5-5.1	4.1 (3.8-4.6)
Chloride, mEq/L	98-107	102 (98-106)
Bicarbonate, mEq/L	22-29	20.6 (16.9-23.7)
Liver enzymes		
GGT, U/L	Male: <55; female: <38	62 (37-113)
ALT, U/L	Male: <45; female: <34	32 (20-51)
ALP, U/L	40-150	95 (73-138)
Blood coagulation indicators		
PT, sec	11-60	11.0 (10.5-12.0)
INR	-	1.05 (0.99-1.16)
D-dimer, µg/ml FEU	Up to 0.5	1.28 (0.61-2.78)
RBG, mg/dL	<140	191 (138-278)
HbA1C, %	Diabetic = ≥6.5	6.8 (6.0-8.8)
Cardiac and inflammatory markers		
HS-cTnI, ng/L	Cutoff 99th percentile: male: 34.2; female: 15.6	23 (7-132)
C-reactive protein, mg/L	<5	116.1 (57.5-190.1)
Serum ferritin, ng/ml	Male: 20-250; female: 10-120	730.9 (340.7-1,488.2)
LDH, U/L	125-220	495.0 (352.5-707.6)
Procalcitonin, ng/ml	<0.046	0.29 (0.11-0.99)

Regarding liver enzymes, the median gamma-glutamyl transferase level was slightly elevated, 62 (37-113) U/L. In contrast, the median alanine transaminase level was slightly low, 32 (20-51) U/L.

Regarding the blood coagulation indicators, the median D-dimer level was elevated (1.28 (0.61-2.78) µg/ml). In terms of blood sugar indicator, the median baseline random blood glucose level and median HbA1C were significantly higher than the normal ranges, 191 (138-278) mg/dL and 6.8% (6.0-8.8%), respectively. These levels indicate poor control of blood glucose levels.

Based on the results, the median high-sensitivity cardiac troponin-I was slightly high, 23 (7-132) ng/L, suggesting potential cardiac stress. Whereas the inflammatory markers were considerably elevated, median C-reactive protein was 116.1 (57.5-190.1) mg/L, serum ferritin was 730.9 (340.7-1,488.2) ng/ml, lactate dehydrogenase was 495.0 (352.5-707.6) U/L, and procalcitonin was 0.29 (0.11-0.99) ng/ml. These elevated inflammatory markers indicate substantial systemic inflammation.

Prescribed treatment during the hospital stay

Antihypertensive therapy for COVID-19 patients with HTN is described in Table 4. Around four-fifths of patients, totaling 270 (81.1%), were prescribed antihypertensive therapy. A total of 157 (47.1%) received calcium channel blockers, 76 (22.9%) beta-blockers, and 75 (22.5%) diuretics during their hospital stay. On the other hand, a limited number of patients - 31 (9.3%) and 7 (2.1%) - received angiotensin-converting enzyme inhibitors and angiotensin receptor blockers, respectively.

**Table 4:** Prescribed treatment during hospitalization (N = 333) * Alpha blockers and ivabradine

Variables	N (%)
Antihypertensive therapy	270 (81.1)
Antihypertensive class	
Calcium channel blocker	157 (47.1)
Vasodilators	85 (25.5)
Beta-blockers	76 (22.9)
Diuretics	75 (22.5)
Angiotensin-converting enzyme inhibitors	31 (9.3)
Angiotensin receptor blockers	21 (6.3)
Other*	7 (2.1)
Antihypertensive treatment scheme	
Monotherapy	140 (42.0)
Dual therapy	80 (24.1)
Multiple therapy	50 (15.0)
Antiviral	254 (76.3)
Antibiotics therapy	184 (55.3)
Corticosteroids	291 (87.4)

Based on the antihypertensive treatment scheme, around two-fifths of patients (42.0%) received monotherapy, followed by dual antihypertensive therapy (24.1%), and multiple antihypertensive therapies (15.0%).

Apart from antihypertensive therapy, patients were also prescribed antiviral, antibiotics, and corticosteroid therapy during hospitalization. Over three-fourths of patients (76.3%) received remdesivir, 55.3% received antibiotics, and 87.4% received corticosteroids.

Complications of patients

Table 5 suggests that patients’ most frequent complication was cardiac arrest (28.8%), followed by multiple organ dysfunction syndrome (8.7%) and acute respiratory distress syndrome (ARDS) (6.3%). Moreover, 44.1% of COVID-19 patients with HTN died during hospital stay.

**Table 5:** Health complications of patients during hospitalization (N = 333) ARDS, acute respiratory distress syndrome

Variables	N (%)	
		Complications		
Cardiac arrest	96 (28.8)	
ARDS	21 (6.3)	
Acute kidney injury	1 (0.3)	
Sepsis	15 (4.5)	
Non-ST elevated myocardial infarction	19 (5.7)	
Multiple organ dysfunction syndrome	29 (8.7)	
Death	147 (44.1)	

Predictors of severity using binary logistic regression

A binary logistic regression model was used to determine the severity predictors among hypertensive COVID-19 patients. Initially, a univariable binary regression model was used by including 35 key characteristics as the independent variable and severity as the dependent variable. Severity levels were categorized as severe and non-severe. Before fitting the binary logistic regression model, continuous variables were converted into categorical variables according to their reference ranges [13], and non-severe illness was fitted as the reference level in the univariable model [14], as given in Table 6.

**Table 6:** Univariable analysis for risk factors of severity (N = 333) ALP, alkaline phosphatase; ALT, alanine transaminase; RBG, random blood glucose; TLC, total leucocyte count

Variable	Severity		Crude OR (95% CI)	p-value
	Non-severe (N = 52)	Severe (N = 281)		
Gender				
Female	32 (61.5)	150 (53.4)	1	0.279
Male	20 (38.5)	131 (46.6)	1.39 (0.76-2.56)	
Age groups				
<65 years	30 (57.7)	156 (55.5)	1	0.772
≥65 years	22 (42.3)	125 (44.5)	1.09 (0.60-1.98)	
Comorbidities				
Diabetes mellitus	30 (57.7)	158 (56.2)	0.94 (0.51-1.71)	0.845
Cardiovascular disease	16 (30.8)	77 (27.4)	0.84 (0.44-1.61)	0.619
Chronic kidney disease	5 (9.6)	31 (11.1)	1.16 (0.43-3.15)	0.763
Signs and symptoms				
Fever	33 (63.5)	246 (87.5)	4.04 (2.07-7.87)	<0.001
Shortness of breath	27 (51.9)	246 (87.5)	6.50 (3.40-12.45)	<0.001
Productive cough	10 (19.2)	119 (42.3)	3.08 (1.48-6.39)	0.002
Chest pain	10 (19.2)	22 (7.8)	0.36 (0.16-0.81)	0.013
Systolic blood pressure				
≤140	26 (50.0)	127 (45.2)	1	0.523
>140	26 (50.0)	153 (54.8)	1.21 (0.67-2.19)	
Diastolic blood pressure				
<90	41 (78.8)	212 (75.5)	1	0.598
≥90	11 (21.2)	69 (24.5)	1.21 (0.59-2.48)	
Heart rate				
<80	18 (34.6)	57 (20.3)	1	0.002
≥80	34 (65.4)	224 (79.3)	2.51 (1.42-4.73)	
Hemoglobin				
Normal	31 (59.6)	191 (68.0)	1	0.242
Abnormal	21 (40.4)	90 (32.0)	0.696 (0.379-1.278)	
TLC				
Normal	24 (46.1)	117 (41.6)	1	0.545
Abnormal	28 (53.9)	164 (58.4)	1.201 (0.663-2.177)	
Neutrophils				
Normal	11 (21.1)	22 (7.8)	1	0.005
Abnormal	41 (78.9)	259 (92.2)	3.159 (1.426-6.996)	
Lymphocytes				
Normal	11 (21.1)	28 (10.0)	1	0.024
Abnormal	41 (78.9)	253 (90.0)	2.424 (1.121-5.244)	
Monocytes				
Normal	40 (76.9)	229 (81.5)	1	0.443
Abnormal	12 (23.1)	52 (18.5)	0.757 (0.371-1.542)	
Eosinophils				
Normal	13 (25.0)	27 (9.6)	1	0.003
Abnormal	39 (75.0)	254 (90.4)	3.136 (1.492-6.589)	
Basophils				
Normal	46 (88.5)	256 (91.1)	1	0.548
Abnormal	6 (11.5)	25 (8.8)	0.749 (0.291-1.926)	
High-sensitivity troponin I				
Normal	39 (75.0)	156 (55.5)	1	0.01
Abnormal	13 (25.0)	125 (44.5)	2.40 (1.23-4.70)	
C-reactive protein				
Normal	6 (11.5)	7 (2.5)	1	0.005
Abnormal	46 (88.5)	274 (97.5)	5.106 (1.642-15.873)	
D-dimer				
Normal	20 ()	43 (15.3)	1	<0.001
Abnormal	32 (11.9)	238 (84.7)	3.459 (1.813-6.601)	
Serum ferritin				
Normal	12 (27.3)	32 (11.4)	1	0.025
Abnormal	40 (13.8)	249 (88.6)	2.334 (1.111-4.906)	
Lactate dehydrogenase				
Normal	11 (50.0)	11 (3.9)	1	<0.001
Abnormal	41 (13.2)	270 (96.1)	6.585 (2.683-16.165)	
Procalcitonin				
Normal	3 (42.9)	4 (1.4)	1	0.064
Abnormal	49 (15.0)	277 (98.6)	4.240 (0.920-19.530)	
Gamma-glutamyl transferase				
Normal	28 (53.8)	104 (37.0)	1	0.024
Abnormal	24 (46.2)	177 (63.0)	1.986 (1.093-3.606)	
ALT				
Normal	32 (61.5)	182 (64.8)	1	0.655
Abnormal	20 (38.5)	99 (35.2)	0.870 (0.473-1.602)	
ALP				
Normal	47 (90.4)	212 (75.4)	1	0.023
Abnormal	5 (9.6)	69 (24.6)	3.059 (1.170-8.000)	
RBG				
Normal	14 (26.9)	73 (26.0)	1	0.887
Abnormal	38 (73.1)	208 (74.0)	1.050 (0.538-2.048)	
HbA1C				
Normal	28 (53.8)	119 (42.3)	1	0.127
Abnormal	24 (46.2)	162 (57.7)	1.588 (0.877-2.878)	
Creatinine				
Normal	36 (69.2)	165 (58.7)	1	0.157
Abnormal	16 (30.8)	116 (41.3)	1.582 (0.838-2.985)	
Sodium				
Normal	20 (38.5)	143 (50.9)	1	0.102
Abnormal	32 (61.5)	138 (39.1)	0.603 (0.329-1.105)	
Potassium				
Normal	41 (78.8)	227 (80.8)	1	0.746
Abnormal	11 (21.2)	54 (19.2)	0.887 (0.428-1.837)	
Chloride				
Normal	27 (51.9)	163 (58.0)	1	0.416
Abnormal	25 (48.1)	118 (42.0)	0.782 (0.432-1.415)	
Bicarbonate				
Normal	19 (36.5)	85 (30.2)	1	0.370
Abnormal	33 (63.5)	196 (69.8)	1.328 (0.715-2.466)	

Table 7 presents the results of the multivariable logistic regression model to identify confounding risk factors for predicting severe illness among hypertensive COVID-19 patients. The variables with a significance of p < 0.05 in univariable logistic regression or relevant to the literature (gender, age, diabetes mellitus, cardiovascular disease, and chronic kidney disease) were analyzed using a backward multivariable logistic regression. The overall significance of the model was confirmed by the likelihood ratio test (X2 = 85.936, p < 0.001), suggesting that the predictors reliably distinguished between severe and non-severe cases. The model explained a moderate amount of the variance, as indicated by the Cox & Snell R Square (R2 = 0.221) and Nagelkerke R Square (R2 = 0.382). The Hosmer-Lemeshow test suggested that the model fits the data well (chi-square = 4.847, df = 7, p = 0.679).

**Table 7:** Multivariable analysis of risk factors of severity ALP, alkaline phosphatase; HS-cTnI, high-sensitivity cardiac troponin-I; LDH, lactate dehydrogenase

Variables	Adjusted OR (95% CI)	p-value
Fever	2.85 (1.18-6.86)	0.019
Shortness of breath	6.23 (2.82-13.77)	<0.001
Chest pain	0.36 (0.13-0.99)	0.049
Pulse rate, >80 bpm	2.63 (1.28-5.42)	0.009
HS-cTnI, >34 ng/ml	2.52 (1.09-5.74)	0.029
D-dimer, >0.5 µg/ml	4.01 (1.82-8.82)	<0.001
LDH,>220 U/L	4.09 (1.31-12.84)	0.016
ALP, >150 U/L	2.76 (0.94-8.17)	0.660

In multivariable analysis, the predictors for severe illness were fever (aOR, 2.85, 95% CI 1.18-6.86, p = 0.019), shortness of breath (aOR 6.23, 95% CI 2.82-12.77; p < 0.001), pulse rate, >80 (aOR 2.63, 95% CI 1.28-5.42; p = 0.009), HS-cTnI, >34 (aOR 2.52, 95% CI 1.09-5.74; p = 0.029), elevated D-dimer, >0.5 (aOR 4.01, 95% CI 1.82-8.82; p < 0.001), and elevated LDH, >220 (aOR 4.09, 95% CI, 1.31-12.84, p = 0.016), keeping all other variables constant, as shown in Table 7.

## Discussion

The COVID-19 pandemic is producing a wide-ranging impact on patients with comorbidities, including HTN. Patients without any history of cardiovascular problems also face palpitations and chest pain complaints after being infected with COVID-19 [15]. To better understand the disease in hypertensive patients, it is essential to determine the predictors of severe illness among hypertensive COVID-19 patients. By doing so, the relevant resources can be appropriately allocated to patients with a high-severity risk and for effective disease management.

While considering the clinical endpoint of the disease, the association between demographic characteristics and the severity of COVID-19 patients with HTN was determined. The results did not show any difference in the severity of COVID-19 patients with HTN based on gender, median age, smoking status, COVID-19 vaccine status, and comorbidities. These findings are consistent with a previous study conducted in China [16], which showed no significant association between severity and demographic characteristics with comorbidities in COVID-19 patients.

The clinical presentation of COVID-19 patients with HTN varies with the severity of the cases. However, the current study showed a significant correlation between clinical presentation and severe illness. Moreover, critical patients reported a higher incidence of shortness of breath, fever, productive cough, and chest pain than patients with severe or non-severe illness, supported by a multicenter study from Malaysia [17]. In addition, we also found that baseline vital signs, including heart rate, respiratory rate, and oxygen saturation, were also associated with the severity of COVID-19 patients with HTN. Furthermore, COVID-19 patients with HTN had even higher median respiratory and heart rates than the respiratory and heart rates reported by Sim et al. [17]. These findings indicate that the SARS-CoV-2 virus mainly affects the respiratory tract and causes lung damage and narrowing of the airway, leading to shortness of breath in severe cases [18], and HTN may further affect the respiratory and cardiovascular systems in patients infected with COVID-19.

Some COVID-19 patients are presented with leukocytosis, associated with neutrophilia, indicating a severe illness [19]. However, with the progression of COVID-19, the neutrophil count gradually increases; therefore, neutrophilia may be considered a marker for severe respiratory illness and poor outcomes [20]. An increasing neutrophil count was identified as a predictor of severe illness among COVID-19 patients [14]. Moreover, Liu et al. identified that the risk of severe or critical illness was higher for patients with neutrophils (>75%), comparable with our results. Neutrophilia reflects the cytokine storm and hyperinflammation associated with the pathogenic mechanism of severe COVID-19, which can lead to collateral tissue damage, exacerbate the severity, and worsen clinical outcomes [21].

In addition, we also found that baseline vital signs, including heart rate, respiratory rate, and oxygen saturation, were also associated with the severity of COVID-19 patients with HTN. The current study observed that oxygen saturation (<90%) was associated with the severity of patients, as reported in a Malaysian study [17]. These findings indicate that HTN may further affect the respiratory and cardiovascular systems of patients infected with COVID-19. Another study from the Netherlands also reiterated that severe COVID-19 patients had lower oxygen saturation, confirming our findings [22]. However, the hypertensive COVID-19 patients in our study posed an even more severe derangement in oxygen saturation. HTN causes structural and functional changes in arteries by narrowing and stiffening them, which reduces the blood flow and oxygen delivery to various organs, including the lungs. In addition, SARS-CoV-2 primarily affects the lungs; therefore, preexisting HTN can further compromise oxygen saturation.

Recent shreds of evidence demonstrated a clear association of elevated HS-cTnI with the severity of COVID-19 [14,23]. Similarly, this study determined that HS-cTnI significantly differed among patients with different severity levels. Patients with critical illness showed the highest values of median HS-cTnI compared to severe and non-severe, reflecting acute cardiac injury due to the direct invasion of SARS-CoV-2 and damage to the myocardium [24].

Research has reported increased D-dimer levels in COVID-19 patients after the onset of inflammation, cell damage, or tissue injury. In addition, elevated D-dimer levels were significantly related to severe illness in COVID-19 patients [25]. Liu et al. found that patients with D-dimer >0.5 had more severe illness than those with D-dimer <0.5, which confirms our results [14]. It may be attributed to a hypercoagulability state, leading to pulmonary embolism or deep vein thrombosis [26], as SARS-CoV-2 may cause damage to blood vessels and clotting. The risk may also be exacerbated due to coexisting HTN.

COVID-19 can cause lung complications during severe illness. Apart from respiratory complications, it may also lead to cardiac complications due to the invasion of SARS-CoV-2 toward cardiac tissues. However, the severity of these complications can vary depending on the individual’s health and COVID-19 severity [14].

Limitations

There are a few limitations to our study. Firstly, being a single-center study, the generalizability of our findings to a larger population may be limited due to the relatively small sample size and non-probability sampling technique, which may potentially introduce selection bias. Secondly, the duration of follow-up was short; therefore, the long-term impact of demographic and clinical characteristics on health outcomes could not be determined. However, only stable patients were discharged from the hospital, which may reduce the risk of bias due to premature discharge and help to ensure that the outcomes observed in our study may represent the broader population of hypertensive COVID-19 patients. Finally, due to the limited sample size, it is possible that some key predictors, such as antidiabetic therapy, may have been missed or not identified sufficiently. Therefore, future studies are warranted to further investigate and validate the predictors in a large population.

## Conclusions

Our study determined that fever, shortness of breath, elevated pulse rate, HS-cTnI, D-dimer, and LDH values are the predictors of severity among hypertensive COVID-19 patients. The study’s findings can be used to inform public health policies and clinical practice in managing COVID-19 patients with HTN in Pakistan and other similar settings. Therefore, healthcare professionals must prioritize hypertensive patients with COVID-19 and provide adequate monitoring and treatment to improve patient outcomes and prevent complications, including cardiac failure, non-ST elevated myocardial infarction, acute kidney injuries, and ARDS.
